# Synthesis of Three-Dimensional Graphene-Based Hybrid Materials for Water Purification: A Review

**DOI:** 10.3390/nano9081123

**Published:** 2019-08-03

**Authors:** Yan Wang, Lei Guo, Pengfei Qi, Xiaomin Liu, Gang Wei

**Affiliations:** 1 College of Chemistry and Chemical Engineering, Qingdao University, Qingdao 266071, China; 2 College of Life Science, Qingdao University, Qingdao 266071, China; 3 College of Materials and Engineering, Qingdao University, Qingdao 266071, China; 4 Faculty of Production Engineering, University of Bremen, D-28359 Bremen, Germany

**Keywords:** fabrication, graphene, three-dimensional, hybrid materials, water purification

## Abstract

Graphene-based nanostructures and nanomaterials have been widely used for the applications in materials science, biomedicine, tissue engineering, sensors, energy, catalysis, and environmental science due to their unique physical, chemical, and electronic properties. Compared to two-dimensional (2D) graphene materials, three-dimensional (3D) graphene-based hybrid materials (GBHMs) exhibited higher surface area and special porous structure, making them excellent candidates for practical applications in water purification. In this work, we present recent advances in the synthesis and water remediation applications of 3D GBHMs. More details on the synthesis strategies of GBHMs, the water treatment techniques, and the adsorption/removal of various pollutants from water systems with GBHMs are demonstrated and discussed. It is expected that this work will attract wide interests on the structural design and facile synthesis of novel 3D GBHMs, and promote the advanced applications of 3D GBHMs in energy and environmental fields.

## 1. Introduction

In recent and coming decades, the lack of clean drinking water is a global concern affecting human living due to the rapid growth of the population, industrial or agricultural pollutions, extended droughts, and fast consumption [1]. It is well known that more than 90% of water in the world is comprised of salty water and only about 2.5% could be used for human consumption, among them most of the water resources are polluted by various industrial dyes, toxic metallic ions, and other aromatic organic chemicals [2]. Traditional polymer membranes have been widely used for water purification [3,4], but they were relentlessly defied by fouling. Because some impurities and biological materials would aggregate on the surface or in the pores of the fabricated membranes, causing very poor selectivity, low water purification ability, reduced resilience, and increased energy consumption [5]. In order to overcome these problems, new techniques to create various materials with low cost, high water purification efficiency, and good antifouling properties for high-performance water purification are required [6].

Many techniques have been utilized for water purifications, in which the adsorption is known as a simple, effective, and economic method to achieve in high-performance water purification due to its high ability to remove various soluble and insoluble organic and inorganic contaminants [7]. In addition, adsorption is capable of retaining contaminates with final concentrations lower than ppb level, thereby achieving higher qualities of water [8]. Pollutants can be removed from water either by adding adsorbents directly to the contaminated water or by employing columns assembled with adequate adsorbents where contaminated water flows through the columns [7]. Adsorption is often adopted for industrial and portable purposes such as source reduction and reclamation. Consequently, adsorptive water treatment has aroused much attention. Typical adsorbents that are frequently used in the pollutants adsorption processes include activated carbon (AC) [9], clay minerals [10,11], zeolites [12], metal oxides [13,14], biomass [15], polymeric materials [16], hydrogels [17], chitosan and its derivatives [18], metal organic frameworks [19] and carbon nanotubes [20]. However, some of the them are expensive and difficult to be separated from the adsorption system as well as cost-ineffective for regeneration [21].

Graphene is functional two-dimensional (2D) sheet material with innovative structural, electrical, mechanical, thermal, and optical properties, which has revealed various applications in nanodevices, energy materials, catalyst materials, biomedical materials, and biosensors [22,23,24,25,26,27,28,29]. 2D graphene oxide (GO) films and membranes have been fabricated by modifying GO nanosheets with various polymers and subsequent self-assembly processes in solution [21,22,23]. Recently, it has been found that the 2D physical structure and adjustable chemical/biophysical properties of GO improved the potential to make function-specific films and membranes by stacking GO sheets for ionic and molecular sieving [30]. The fabrication of large-scale graphene membranes with desired thickness and pores could be achieved by assembling GO in solution through several techniques like vacuum filtration, self-assembly, direct drop casting, and physical coating [31,32]. In addition, the GO sheet is easy to be modified with other chemicals to create well-defined nanoscale pores with low frictional water flow inside, which could be of high interest for water filtration and separation [33,34].

Compared to traditional 2D graphene, three-dimensional (3D) graphene-based hybrid materials (GBHMs) with nanoporous/microporous structure could provide higher porosity and larger specific surface area as well as excellent electrical and thermal conductivity, and therefore, both adsorptive and catalytic performances of 3D GBHMs can be improved significantly [35,36,37,38]. For example, previously, Li et al. presented advances in the synthesis and potential applications of 3D graphene micro-/nano-architectures [35]. Qiu et al. demonstrated recent development of 3D GBHMs for catalytic applications [37], and very recently Wu and co-workers summarized the synthesis strategies of 3D graphene-based materials, which exhibited wide applications in the fields of energy storage applications of [38]. In addition, 3D GBHMs have been found to be highly useful in the fields of water purification and environmental monitoring [39].

We realize that it is important to summarize recent advances in the fabrication of 3D GBHMs, and their potential applications for water purification applications with this work. In Section 2, the methods for fabricating 3D GBHMs, such as the template-based synthesis, self-assembly, ice-drying of hydrogels, and 3D printing, are introduced. Then in Section 3, various techniques for water purification are demonstrated. In Section 4, the fabricated 3D GBHMs for removing metallic ions, anions, organic dyes, drugs and biomolecules, as well as for oil-water separation and seawater deionization, are introduced and discussed in detail. It is expected that the detailed demonstration and extended discussion are helpful for the researchers of materials science to develop novel techniques and functional materials for water purification applications in one way, and in another way, to know the pollutant adsorption and desorption mechanisms of 3D graphene-based materials towards various pollutants in water systems.

## 2. Fabrication Methods of 3D GBHMs

**Figure 1:**
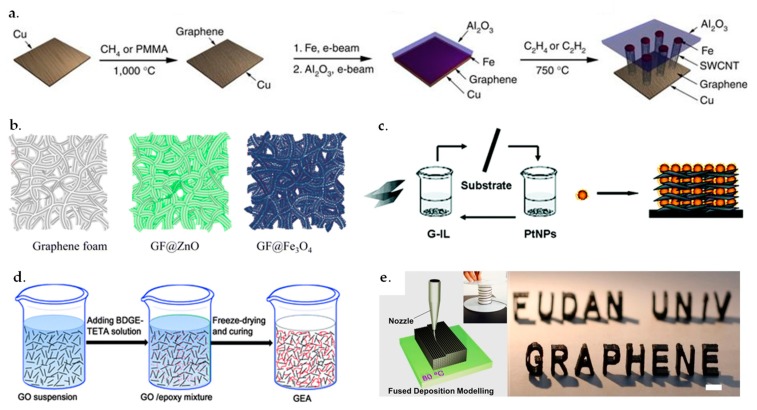
Fabrication strategies of three-dimensional (3D) graphene-based hybrid materials (GBHMs) (**a**,**b**) templated synthesis. Reproduced from [40,47], with permission from Nature Publishing Group, 2012 and American Chemical Society, 2013. (**c**) Self-assembly fabrication. Reproduced from [41], with permission from American Chemical Society, 2010. (**d**) Freeze drying. Reproduced from [42], with permission from Royal Society of Chemistry, 2013. (**e**) 3D printing. Reproduced from [43], with permission from Nature Publishing Group, 2015.

The fabrication of 3D GBHMs can be achieved through several strategies including template-based synthesis [40], self-assembly [41], freeze-drying [42], 3D-printing [43], and others, as shown in Figure 1. In this section, we would like to present the basic mechanisms for the design and fabrication of 3D GBHMs with these methods.

### 2.1. Template-Based Synthesis

Template-based synthesis is a simple but effective method to create 3D GBHMs through conjugating nanoparticles (NPs), polymers, carbon nanotubes (CNTs), and other nanostructures onto 3D graphene substrates. The synthesis of 3D reduced GO (rGO) supported PtM (M stands for Fe, Co, Ni) hollow nanospheres was reported by Qiu and co-workers [44] through a universal sacrificial template-based synthesis strategy. The proposed approach achieved an identical synthesis route for the preparation of hybrid nanomaterials with various metal nanostructures and is considered to be more promising from the environmental point of view as no extra high-temperature reactions and/or toxic solvents were adopted.

In addition, it is possible to use chemical vapor deposition (CVD) to fabricated 3D flexible and conductive graphene networks [45], which can then modified with other nanomaterials to form 3D GBHMs easily [46]. For instance, Cao et al. [46] utilized the facile CVD method to synthesize MoS_2_-coated 3D graphene (MoS_2_/3DGN) composites by employing 3DGN as the template for the direct growth of MoS_2_. The as-prepared MoS_2_/3DGN composite was used as anode material for lithium-ion batteries, which exhibited excellent electrochemical performance. Zhu and co-workers [47] reported a method for the synthesis of graphene and CNT carpet hybrids by firstly forming the graphene onto the surface of Cu foil through CVD, and then depositing Fe and Al_2_O_3_ on the graphene-covered Cu foil through e-beam evaporation, followed by growing CNT carpet directly on the graphene surface. As a result, 3D graphene and CNT carpet hybrids were synthesized. The scheme for the preparation of graphene and CNT carpet hybrid material is illustrated in Figure 1a.

3D GBHMs could also be fabricated by conjugating various nanostructures onto the graphene foams (GFs) based on metal and polymer foams [40,48,49,50]. For example, Luo et al. [40] reported the preparation of 3D GF supported Fe_3_O_4_ hybrids through grafting Fe_3_O_4_ NPs onto a GF substrate, as shown in Figure 1b. This strategy is facile for creating a lot of 3D GBHMs by conjugating different kinds of nanomaterials onto/into GFs. In another case, Chang and co-workers demonstrated the fabrication of 3D GBHMs by growing MoSx onto the graphene-protected 3D Ni foams [48]. It was found that the modification of Ni foams with graphene sheets provided robust protection for the 3D materials and enhanced the stability of materials in acidic systems, exhibiting high potential for electrocatalytic hydrogen production.

### 2.2. Self-Assembly

Self-assembly is identified as a promising strategy for incorporating various nanostructured materials into macroscopic substances [51]. It is capable of transforming features of nanostructures to the resulting macroscopic materials with hierarchical structures and novel functionalities [52]. In addition, the assembled superstructures possess some astonished physiochemical properties that are different from any individual components, allowing expanded capacities for real applications [51]. Recently, it has been found that graphene nanosheets can be utilized to fabricate 3D scaffolds/networks via a self-assembly strategy, in which the addition of other nanoscale building blocks can mediate the formation of 3D GBHMs. Self-assembly strategies, including hydrothermal reduction process [53], metal ion induced process [51] and chemical reduction process [54] make graphene sheets interconnect with each other through electrostatic interactions, π–π stacking and hydrogen bonding [55].

For example, Zhu et al. adopted a layer-by-layer self-assembly method to create a 3D graphene/Pt NPs nanocomposites by employing ionic liquids (ILs) as the linkers [41], as shown in Figure 1c.

Firstly, GO nanosheets were modified with ILs to generate a positively charged surface onto indium tin oxide (ITO) surface, which was then immersed into a negatively charged Pt NPs solution for electrostatic binding to form GO-IL/Pt NPs hybrids. The ordinary layer-by-layer self-assembly made it possible to fabricate 3D GBHMs after a switch of GO to graphene through thermal reduction. Ye et al. proposed the synthesis of 3D hierarchical graphene/polypyrrole nanotube hybrid aerogel by a simple reduction self-assembly process [56]. It was found that the polypyrrole nanotubes mediated the self-assembly of the graphene sheet by providing a huge approachable surface area, which are severed as spacers to inhibit the aggregation of graphene. Zhu and co-workers introduced a facile hydrothermal reaction-based self-assembly strategy to fabricated a graded rGO/MnO_2_ hybrid hydrogel [57]. In another case, Wu and co-workers developed a facile route to prepare 3D graphene/polymer composites by using the GO-polymer electrostatic interaction-caused self-assembly process [58].

### 2.3. Freeze-Drying

Freeze-drying has attracted considerable attention among various methods developed for the fabrication of porous materials, as it is a versatile, easily accessible and low-cost phase segregation process that can make use of the controlled crystallization of a solution to produce ordered hierarchical porous structures [59]. Recent published work has demonstrated that 3D porous GBHMs can be fabricated by freeze-drying technique, in which the mechanical strength of 3D GBHMs could be improved by adding linkers to connect graphene sheets [42,60,61]. For example, Vickery and co-workers demonstrated the fabrication of 3D graphene-polyvinyl alcohol (PVA) nanocomposites with high order 3D structures with freeze-drying [62]. Ye and co-workers reported the synthesis of high-elastic GO-epoxy aerogels by freeze-drying and curing [42], as shown in Figure 1d. The resulting 3D network GBHMs exhibited high decomposition temperature, excellent mechanical strength, low density, and very high elasticity. In another case, Lu et al. indicated that the freeze-drying was effective to fabricate 3D S-graphene hybrid sponges, which showed potential application for lithium-sulfur batteries with high regional mass load [63].

In addition, 3D GBHMs by conjugating chitosan, GO, and hydroxyapatite nanocomposites have been fabricated by the freeze-drying synthesis and then used for biomedical application [64].

### 2.4. 3D Printing

3D printing is a novel fabrication technique to create various 3D structured materials, offering a distinct pathway for fast prototyping of numerous applications owing to its capability of producing inexpensive 3D printed platforms [65]. It can also be utilized to fabricate different 3D graphene materials and GBHMs [43,66,67,68]. 3D printing bridges the gap between graphene materials and the digital mainstream, which promotes the graphene revolution into a new step [67].

For example, Foster and co-workers [65] reported the fabrication of a number of 3D disc electrode architectures that were utilized for energy storage devices. As the effective electrode material, 3D graphene-based polylactic acid filaments were produced by using a RepRap fused deposition molding 3D printer. Zhu et al. proposed the fabrication of 3D periodic graphene aerogel (GA) microlattices by using the 3D printing approach [66]. The direct ink writing could produce 3D GAs with unique properties like lightweight, highly conductive, and supercompressibility. In a further study, they produced 3D hierarchical GAs with regular macropores with a 3D printing technique, which can be used for high-performance supercapacitors by introducing graphene NPs into the system [68]. In another case, Wei et al. [43] demonstrated a graphene/acrylonitrile-butadiene-styrene (ABS) composite that could be 3D printed into computer-designed models, as shown in Figure 1e. The printing process was easy, rapid, and effective to achieve in exotic topological structures. The synthesis of graphene-polymer composites via the solution-based 3D printing process can be readily scaled-up for industrial applications.

## 3. Techniques for Water Purification

Nowadays, water is being purified by various approaches, but studies have been persistently conducted to look for reliable and effective advanced materials for water purification at a considerable level. In this section, we introduced and discussed current advances of available technologies and the corresponding materials for water purification. In particular, the 3D GBHMs that have been involved in these water purification techniques are briefly discussed.

### 3.1. Filtration

The filtration of water systems is based on passing water through a filter (e.g., a porous medium), employed to eliminate suspended solids from water. Filtration is often applied for processing various source of waters for both industrial and civilian applications. With the developments in filtration technology in the past decades, various types of filtration systems have gained increased popularity. For water treatment, typical filtration systems are gravity-based filtrations such as rapid gravity filtration and slow sand filtration, and pressure-based filtrations including rapid rate pressure filtration and membrane filtration. Among them, membrane filtration is identified as one of the most popular technique towards water treatment due to its demonstrated superiorities such as easy operation, energy saving, and high efficiency [69].

Polymetric and inorganic materials including ceramics and metallic oxides have been adopted to fabricate different filtration membranes in the past decades, however, the majority of them are restrained by the contradiction between selectivity and permeability and were often encountered with high costs of fabrication [70]. Besides, other potential materials are still being explored to join the filtration membrane community. Recently developed nanostructure materials such as metal organic frameworks (MOFs) and carbon-based nanocomposites, possess great opportunities as promising membrane materials, which have exhibited superior separation performance and reliable processing capabilities [70]. Among various membrane materials, GBHMs, by incorporating graphene and its derivatives with those functional materials (usually inorganic matter), have received much attention in membrane filtration applications [71,72,73,74]. The synthesized inorganic-polymer hybrid membranes incorporate the excellent film forming ability of polymers and the particular features of inorganic fillers, as well as introduce some surprising properties [74,75]. By integrating GO into various polymer frameworks, the fabricated hybrid ultrafiltration membranes exhibited improved antifouling capabilities and higher membrane permeate fluxes than the pristine polyvinylidene fluoride (PVDF) membranes [76]. Besides, by the incorporation of GO and polyethylene oxide, the ionic conductivity of electrolyte membrane can be significantly increased [77]. A recent work reported the fabrication of two types of membranes by adding GO and rGO nanosheets into sodium alginate (SA) matrix, respectively, through physical blending [71]. The as-prepared membranes exhibited entitative morphology and enlarged free volume, allowing excellent separation performance and high permeate flux to be achieved in membrane filtration water treatment. Also, enhanced mechanical stability and resistance to swelling caused by the mutual effect of SA matrix and GO nanosheets, endow the synthesized membranes good long-term operation stability.

Recently, 3D crumpled GBHMs containing functional NPs emerged as promising water treatment membranes [78]. Different from 2D flat GO analogues, crumpled 3D GO based materials are aggregation-resistant and enable the integration of multifunctional particles (e.g., TiO_2_ and Ag NPs) inside their 3D composite structures, allowing much improved water flux and filtration efficiencies towards organic and biological pollutants in wastewater as compared to those commercial ultrafiltration membranes [79].

All these demonstrated outstanding physical and chemical properties, superior mechanical strength and low fabrication cost, allow GBHMs as membranes to be applied in water purification industries.

### 3.2. Adsorption and Removal

The adsorption technique is the most frequently studied and industrially adopted approach [21]. Adsorption is defined as the concentration increase of a specific substance at the surface and/or interface of two phases [80]. During adsorption, adsorbates are attracted by an adsorbent through physical and/or chemical effects. Generally, adsorption depends on various factors including temperature, pH of solution, operation time, pollutants concentration, nature of adsorbates and adsorbents, etc. [81].

As one of the most prevalent adsorbents, AC has been widely used in water purification community due to its porous structure, specific surface activity, as well as high chemical and thermal stability [21]. However, AC is cost-ineffective and very hard to be removed from aqueous solution with its powdered form. Also, the regeneration of exhausted AC through thermal and/or chemical process is tedious and costly, and often induces loss of the sorbent [21]. In the past years, new adsorption processes have been explored for the removal of various kinds of contaminants from wastewater, while a large variety of approaches have been employed for exploiting high-efficient and low-cost composite adsorbents [21]. Composite materials combine the merits of each component, which are considered as promising alternative as adsorbents since they possess high reactivity and selectivity towards specific pollutants in wastewaters. In particular, graphene-based composites with 3D structures as ideal adsorbents for the elimination of different kinds of contaminants in water systems, have attracted tremendous research interest, owing to their huge specific surface areas, strong electron transport capacities and high mechanical strengths [35,82,83,84,85]. For example, Xu and co-workers [86] reported the preparation of GO/DNA composite hydrogels via a facile 3D self-assembly technique and adopted them for dyes adsorption. The as-prepared GO/DNA composite hydrogels possessed high environmental stability and mechanical strength, excellent dye-adsorption capability, and astonished self-healing property. Bi et al. [87] prepared the spongy graphene (SG) through a hydrothermal treatment approach and utilized it for the selective adsorption of organic solvents and oil, respectively. The synthesized SG showed significantly improved adsorption efficiency towards petroleum products and toxic solvents than that of the previously reported adsorptive materials such as planarization polymers and expanded graphite without any further pretreatment. Moreover, SG can be regenerated more than ten rounds via thermal treatment with an almost complete release of adsorbates each round. Guo and co-workers [84] reported a preparation of the 3D Fe_3_O_4_-graphene macro-composites for the extremely low concentrations of As^3+^ and As^5+^ adsorption from aqueous solution. The combination of graphene macroscopic gel with Fe3O4 by employing polydopamine strengthened 3D graphene-based macroscopic architecture and improved the binding capacity and loading amount of Fe_3_O_4_ nanoparticles. As a result, the 3D Fe_3_O_4_-graphene macro-composites are capable of effectively eliminating sub-ppm concentrations of the arsenic and could be readily separated from aqueous solution by a filter.

All these studies demonstrated the superior adsorption performance of 3D GBHMs in water purification, outperforming majority of the conventional adsorbents.

### 3.3. Photocatalytic Degradation

Many chemicals with high toxic such as pesticides, dyes and detergents are commonly found in industrial wastewater. Nevertheless, these contaminated wastewaters cannot be effectively purified by the prevailing water treatment methods [1,88]. Photocatalytic degradation, with its capability of decomposing many organic contaminates into innocuous carbon dioxide and water through a bunch of oxidation and reduction reactions that are aroused by photo-excited electrons and holes under the irradiation of UV or sunlight, is considered as a promising alternative for wastewater treatment [88,89,90,91].

**Figure 2:**
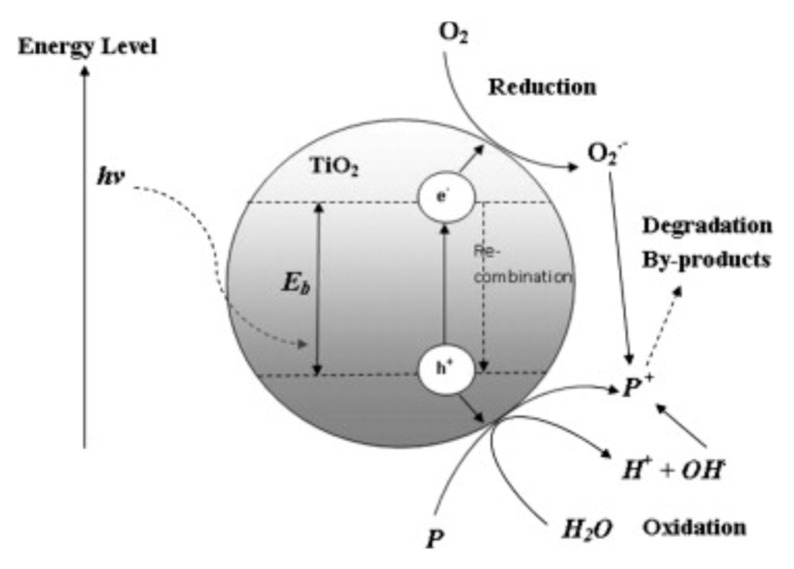
Mechanism of the electron-hole pair formation and the pollutant (P) degradation. Reproduced from [92], with permission from Elsevier, 2010.

In the past decades, photocatalytic degradation has exhibited huge potentials as a green, sustainable, and cost-effective wastewater treatment process with null waste emission [92]. During the photocatalytic degradation process, photocatalysts, i.e., semiconductors, are used to accelerate the chemical reaction and they can be activated by light illumination [93]. Semiconductor catalysts such as Fe_2_O_3_, ZnO, ZnS and TiO_2_ are often employed in photocatalytic degradation, which have been verified to be capable of degrading a huge variety of persistent organic compounds and microorganisms in water. Among them, TiO_2_ has gained the greatest attention in photocatalytic degradation, owing to its distinguished features such as non-toxicity, strong activity at 300–390 nm photon energy range, low-cost availability and high chemical stability even after repeated catalytic cycles [92,94,95]. As a photocatalyst, TiO_2_ is capable of inducing a series of oxidation and reduction reactions on its surface since the outer orbital of TiO_2_ possesses unique lone electron features. The lone electron can be instantaneously excited to the empty conduction band as long as the photon energy (*hv*) is illuminated onto the surface of TiO_2_ [92]. The electron-hole pair (e^−^-h^+^) is thus generated due to a remaining empty unfilled valence band induced by the photonic excitation. The mechanism of the electron hole-pair formation is illustrated in Figure 2. Organic pollutants can either be directly oxidized by the hole itself, or be indirectly oxidized by hydroxyl and superoxide radicals that are created through the mutual effect of holes with water and oxygen [96,97]. As a result, oxidation and reduction reactions can occur at the TiO_2_ surface. Photodegradation of organic contaminates that happens in the presence of TiO_2_ can be expressed as follows [92]:

(1) $$\mathrm{Organic}\;\mathrm{Contaminates}\overset{\;{\mathrm{TiO}}_{2}/hv\;}{\to }\mathrm{Intermidiates}\to {\mathrm{CO}}_{2}+H_{2}O$$

In Equation (1), with the presence of the photocatalyst TiO_2_ and adequate photon energy, the organic contaminates in wastewater are degraded to the corresponding intermediates, and finally transformed into CO_2_ and H_2_O.

However, despite the great potential of TiO_2_ NP as a promising photocatalyst, its application in industrial scale is hampered by the following two obstacles: One is that in the visible range of the solar spectrum, TiO_2_ has relatively low photocatalytic activity; the other is that the fast regroup of the created TiO_2_ electron-hole pairs results in the low efficiency of competing photo-induced chemical reactions [98,99]. An alternative approach to overcome the afore-mentioned problems is the integration of TiO_2_ into graphene (e.g., GO or rGO) to form a graphene/TiO_2_ composite photocatalyst. This is because graphene has higher electron mobility, it accelerates the transmission of the electron, thereby the recombination of electron-hole pairs can be effectively restrained [100]. Besides, with the introduction of graphene, the agglomeration of TiO_2_ NP is thus eliminated. As a result, pollutants get more active sites for degradation due to the maintained large specific surface area, allowing improved photocatalytic degradation performance with a less amount of photocatalysts being consumed. Moreover, doped graphene/TiO_2_ photocatalysts possess much improved photocatalytic activity towards pollutants degradation in the visible range of the solar spectrum resulting from increased extended visible light absorption [100]. Further, the intercalation of TiO_2_ into GO accelerates the sedimentation of photocatalysts compared to pristine TiO_2_ NP and thus facilitates the subsequent catalyst separation and recycling process, which is a benefit for large-scale water purification [101]. For example, Jiang et al. [102] reported the synthesis of crumpled graphene-TiO_2_-magnetite (GOTIM) ternary nanocomposites as multifunctional photocatalysts for water remediation. The as-prepared monomeric, aggregation-resistant GOTIM exhibited much improved photocatalytic properties (at least 20-fold performance enhancement) as compared to the pristine TiO_2_, allowing significantly expanded photocatalytic application potential. For the photocatalytic degradation of pollutants in wastewater by employing TiO_2_ photocatalysis with GO and rGO modifications, a list of studies have been extensively reported [103,104,105,106,107].

### 3.4. Electrocatalytic Degradation

The electrochemical method for organic pollutants degradation in wastewater treatment has received a great deal of attention in recent years, mainly due to its high efficiency, universal applicability and environmental sustainability [108,109]. The mechanism of the electrocatalytic gradation of organic contamination in water is based on the electrocatalytic oxidation process, in which a superoxidative hydroxyl radical is generated on the anode of the electrode that is driven by a power supply [110,111].

During the electrocatalytic process, the electrode material is a crucial factor in improving the degradation efficiencies and optimizing the electrochemical oxidation conditions. Many researchers investigated the electrode development, and various electrode materials have been studied previously, including metal/metal oxide material such as Pt, PbO_2_, and SnO_2_, and carbon-based material such as graphite, carbon fiber, and AC. Among them, metal oxide PbO_2_ is favored as an electrocatalytic material in view of its good electrical conductivity, large oxygen overpotential, and chemical inertness in applications for electrolysis, electrosynthesis, and more recently for wastewater treatment [112]. It was found that as nonactive electrodes, PbO_2_ possessed better organic pollutants degradation performance, comparing to that of the active electrodes (e.g., Pt electrode) [113]. Nevertheless, during the electrochemical degradation of organic pollutants in aqueous solution, electrode fouling frequently happens since the polymer products deposit on the surface of PbO_2_ electrodes and thus decrease the efficiency of the electrocatalytic gradation [114,115]. Treatment of pollutants in a high acidic medium is considered a possible solution to eliminate the electrode fouling problem. In view of this, Zhou and co-workers reported a β-PbO_2_ electrode for the electrocatalytic degradation of phenolic wastewater. To enhance the electrode stability in acidic media, the PbO_2_ electrode was modified by co-depositing fluorine resin on ceramic media [115,116]. This modified β-PbO_2_ electrode was then testified to be capable of degrading phenolic compounds in water partially and selectively, as the activities of reaction were varied with different substituents [115]. In other studies, modified and/or doped metal oxide electrode materials such as RuO_2_/Ti and Sb-SnO_2_/Ti [117], Fe_2_O_3_-modified kaolin [118] as well as multilayered Ti/SnO_2_ + Sb_2_O_3_/MnO*_x_* materials as anodes have been developed towards improving electrocatalytic degradation performance of specific organic compounds in wastewater and their electrochemical behaviors are investigated.

Carbon-based electrode material such as AC has been used as particle electrode for many years. Novel porous carbon materials like carbon aerogel (CA) [119,120] and GA [121,122] with 3D structures were applied to the processing of various pollutants in wastewaters as ideal particle electrodes for electrocatalytic oxidation, and have been proved to exhibit superior electrocatalytic degradation performance to organic pollutants in view of their large specific surface area, excellent electrical conductivity and good chemical stability. Very recently, a new 3D electrode system assembled with nitrogen-doped GA particle electrodes was reported for wastewater treatment that contains Bisphenol A (BPA) through electro-catalytic oxidation [122]. The proposed 3D electrode was demonstrated to be capable of degrading pollutants much more efficiency than that of commercial carbon electrodes and traditional 2D electrodes. Other GBHMs that have been successfully applied as electrocatalysts and/or electrodes for the oxidative degradation of organic contaminants in wastewater can be found elsewhere [123,124,125,126,127].

### 3.5. Biodegradation

Biodegradation is identified as a reduction in complexity of the chemical compound caused by biological catalysis [128]. In fact, it is a process in which organics are transformed into innocent substances in the presence of microbial organisms [129]. As a typical biological water treatment technique, biodegradation has received popularity to remove toxic and noxious pollutants from wastewater due to increased performance, availability and low cost of raw materials [130]. Biological materials, including living and non-living microbial organisms, i.e., bacteria, algae, yeasts, fungi and protozoa, are often employed in biodegradation process towards wastewater treatment to degrade (or mineralize) organic pollutants to innocuous substances such as carbon dioxide, water and bacterial cells through metabolic or enzymatic processes [131]. Organic contaminates can be degraded either aerobically in the presence of O_2_ or anaerobically in the absence of O_2_.

For instance, Hatzinger [132] summarized several ex-situ and in-situ applications for cleaning perchlorate-contaminated groundwater and wastewater via a biodegradation process. Perchlorate reduction process employs perchlorate-reducing organisms by coupling the oxidization of an organic or inorganic electron donor in a form of anaerobic respiration, and produces chloride and oxygen as degradation products. Bernhard et al. [133] studied the non-adsorbing persistent polar pollutants (P^3^) biodegradation, i.e., drugs, pesticides, surfactants and retardants, during wastewater treatment by employing a optimal designed membrane bioreactor (MBR). Comparing to traditional activated sludge treatment, the MBR exhibited significant better removal of poorly biodegradable P^3^, including diclofenac and sulfophenyl carboxylates through aerobic biodegradation, in which P^3^ were assimilated into more universal bacterial path or completely mineralized.

**Figure 3:**
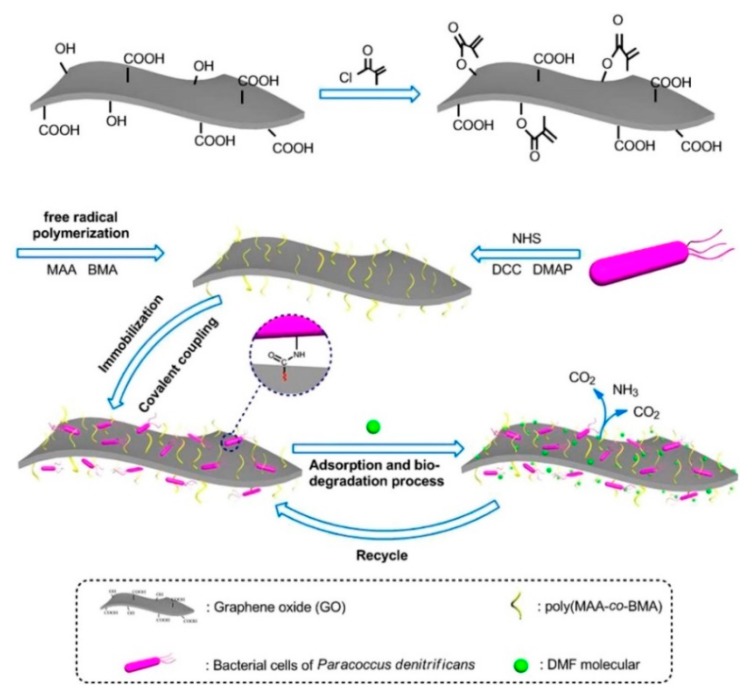
Schematic diagram of PGO synthesis and the adsorption-biodegradation process of the *N*,*N*-dimethylformamide (DMF) molecular on bacterial cells immobilized PGO. Reproduced from [134], with permission from Nature Publishing Group, 2016.

Recent studies showed that the biodegradation performance can be significantly improved by introducing GBHMs in biological water treatment process. Zheng et al. [134] reported the preparation of a new micro composite material polymer GO (PGO) by functionalizing GO with co-polymers including butyl methacrylate (BMA) and methacrylic acid (MAA) through free radical polymerization (Figure 3). The as-prepared PGO was adopted as a high-efficient adsorbent as well as a superior supporter for the paracoccus denitrificans (PD) cells immobilization in treating *N*,*N*-dimethylformamide (DMF) solution at high concentrations. The processes of PGO synthesis and adsorption-biodegradation of DMF molecular on bacterial cells immobilized PGO are illustrated in Figure 3. The proposed PGO@PD hybrid material was testified to be capable of completely removing DMF (2000 mg/L) containing wastewaters. In addition, to remarkable removal efficiency, the PGO@PD hybrid material also showed a much improved recycle performance, compared to that of the pristine PD cells and the PGO material, respectively. In another case, functionalized rGO was adopted for the immobilization of horseradish peroxidase (HRP) onto its surface the through covalent bonding process to improve stability and activity of HRP towards biodegradation of wastewater containing high concentrations of phenol [135]. Verification experiments revealed that both the storage stability and the catalytic activity of the immobilized HRP were greatly improved. As a result, a complete degradation (removal efficiency of 100%) of the high concentration of phenol compound (2500 mg/L) was achieved for the immobilized HRP, comparing to an only 55% removal efficiency for the free HRP.

### 3.6. Capacitive Deionization

Capacitive deionization (CDI), which is based on the electrical adsorption of excessive ions in electrical double layer (EDL) region of the porous electrodes to remove impurities form aqueous solutions under an applied electrical potential, is one of the most promising technology for water purification that has recently gained increasing popularity, especially in seawater desalination community [136]. Comparing to the prevalent water treatment techniques such as membrane filtration and distillation, the benefit of CDI is that it does not need any external heat supply or pressurized equipment, thereby the operation costs can be significantly reduced. In particular, CDI is more fascinating than those traditional techniques for brackish water purification at very low concentrations, as it mainly concentrated on attracting salts in seawater rather than eluting pure water from salt solution [137].

**Figure 4:**
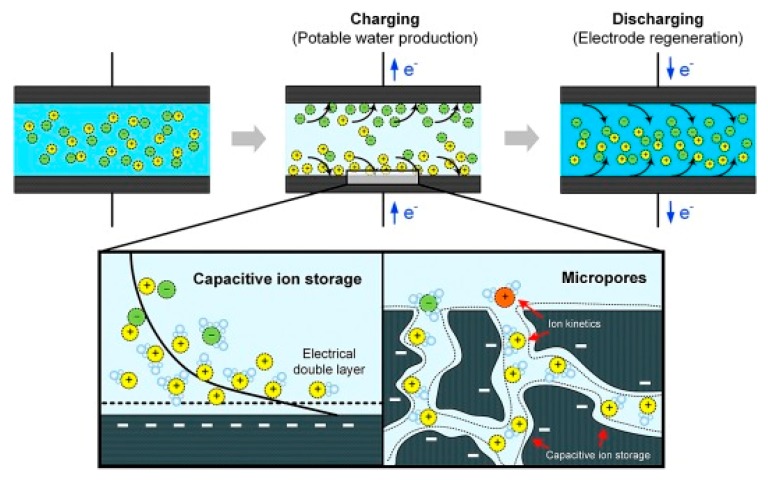
Scheme of the typical capacitive deionization (CDI) process for saline water purification. Reproduced from [139], with permission from Elsevier, 2018.

The CDI process occurs at the charged electrode in the saline water, where ions with opposite charges move towards anode and cathode of the electrode respectively, maintaining the EDLs formation at surfaces of the electrode [137]. Due to electrostatic interaction, cations and anions are adsorbed by the porous electrode, the saline water is therefore deionized. After capacitive deionization, the electrodes can be recycled by short out the electrode or by reducing the applied electric potential of the CDI cell, and the trapped ions can be desorbed back into the solution [138,139]. The main mechanism of ion storage and salt removal during CDI desalination is linked to the non-Faradaic capacitive ion storage process (Figure 4), by which the EDL was formed at the electrode where ions are electrostatically attracted and capacitively reserved at the diffuse layer of the electrode pores, as illustrated in Figure 4 [139,140].

To enhance the non-Faradaic capacitive storage of ions, significant efforts are made towards the fabrication of different kinds of electrode materials as CDI electrodes that possess high adsorption capacity, large surface area, adequate pore size and layout, excellent thermal and electrical conductivity, as well as good chemical and mechanical stability. Generally, CDI electrodes are made of porous carbon materials in various forms including AC, CNTs, mesoporous carbon (MC), graphene, GO, etc. [141,142,143,144,145,146]. However, some of the carbon-based materials suffer from issues such as high fabrication costs, low adsorption capability and poor wettability and stability, which have restricted CDI at scaling-up applications [147]. Recently, carbon-based composite materials that embraced the excellent features of carbon and other constituent materials as CDI electrodes have gained increasing attention. These additional components inside the composite are capable of increasing the electric adsorption capability of carbon materials through modifications of either the pore structures or the surface properties by incorporating some functional groups onto their surfaces [147]. In particular, graphene-based hybrids have been successfully applied as electrode materials for CDI saline water desalination in recent studies [148,149,150,151], and have been verified to possess superior desalination performance with significantly enhanced electrosorption capacity and reduced energy consumption, owing to the strengthened electric conductivity, enlarged specific surface area, as well as more uniform pore layout [152]. Some of the applications on saline water purification by employing the GBHMs as CDI electrode are summarized in the following sections.

## 4. 3D GBHMs for Water Purification Applications

Over the past decades, 3D GBHMs have received increasing attention as they not only reserve the intrinsic properties of graphene, but also inspire astonishing features, exhibiting immense application capability and potential in water purification fields. In fact, the unique structural features as well as superior chemical, electrical, thermal and optical properties endow 3D GBHMs as promising materials for water purification through adsorption, catalysis, filtration, and capacitive deionization [39]. To date, 3D GBHMs have presented excellent application performance in removing environmental pollutants, such as heavy metallic ions, organic pollutants, inorganic anionic pollutants, salts, and others from the wastewater systems. In this section, we expected to summarize recent advances of 3D GBHMs for water purification applications.

### 4.1. Removing Metallic Ions

Metallic ions, especially heavy metal ions, are often found in wastewater discharged from agricultural and industrial fields, which are mostly, highly-toxic. The emission of metallic ions without effective treatment is harmful to the environment as well as the health of human beings. To this end, various techniques have been well established and utilized towards effective removal of various metallic ions from contaminated water. Among them, the adsorptive separation that is based on either physical/chemical adsorptions or ion exchange adsorption is known as the most widely adopted technologies [153,154].

Comparing to conventional adsorbents, 3D GBHMs were found to possess a considerably strong adsorption capability on the elimination of a variety of metal ions. For instance, GO-TiO_2_ hybrids prepared by the self-assembly of TiO_2_ on exfoliated GO were applied for removing heavy metal ions including Cd^2+^, Pb^2+^ and Zn^2+^, from wastewater [155]. It was found that the removal efficiency of these heavy metals can be significantly improved due to mutual effect of the incorporated oxygenated functional groups of the exfoliated GO in combination with TiO_2_ NPs on GO structure. The fabricated GO-TiO_2_ hybrids exhibited the adsorption capacities as high as 68.3, 74.4 and 92.2 mg/g for, Cd^2+^, Pb^2+^ and Zn^2+^, respectively. In addition to GO-TiO_2_, rGO-TiO_2_ hybrid materials also showed improved removal performance of Cr^5+^ from the aqueous solution through adsorption and photocatalytic reduction [156]. A maximum Cr^6+^ removal rate of 86.5% was obtained with rGO-TiO_2_ hybrids when applying with a visible light, much higher than that of 54.2% with pristine TiO_2_. This is primarily attributed to the fact that the addition of rGO enhanced the light absorption strength and reduced the electron-hole pair recombination in TiO_2_. Recently, Tan et al. [157] reported a facile and high-efficient approach for removing uranium (U^6+^) from water by using hierarchical 3D rGO/LDH (layered double hydroxide) composites as the adsorbents. The prepared 3D rGO/LDH was experimentally evaluated towards U^6+^ adsorption with a maximum adsorption capacity of 277.80 mg/g, revealing a remarkable adsorption performance for the U^6+^ removal from wastewater.

Also, the removal of mercury ions Hg^2+^ from wastewater was investigated by employing a 3D porous structure of graphene composite constitute of graphene nanosheets assembled with α-FeOOH and silica microspheres and functionalized by thiol [158]. A very high adsorption capacity for Hg^2+^ (larger than 800 mg/g at 400 mg/L concentration of Hg^2+^) was obtained, which outperforms currently prevalent adsorbents. Meanwhile, astonishing adsorption performances were also achieved for the perfect (up to 100 percent of the removal rate) removal of 120 mg/L (moderate concentration) and 4 mg/L (low concentration) concentrations of Hg^2+^ respectively in water using the proposed composites as membranes.

The applications of 3D GBHMs as adsorbents for metal removal from water are usually restrained as the separation and recycling of these adsorbent materials after the adsorption process are considerably difficult. An alternative way to alleviate this problem is the development of magnetic metal oxide/GO composites, which can be readily separated by using a magnet [159]. Successful adsorption of Co^2+^ [154], Cu^2+^ [160], U^6+^ [161], Cr^6+^ [112], and As^3+^ [84] in aqueous solution by using magnetic Fe_3_O_4_/GO or Fe_3_O_4_/rGO hybrid materials have been demonstrated with satisfactory removal performances. A recent study [162] reported the effective removal of Cr^4+^ from water by a novel 3D bulk Fe_3_O_4_/GO foam synthesized on a Ni foam by oxidizing and magnetically functionalizing with Fe_3_O_4_ NPs. Benefitting from the layered porous structure with a huge specific surface area of 574.2 m^2^/g, 3D Fe_3_O_4_/GO foam presented an excellent absorption performance for removing Cr^4+^ ions with the absorption capacity as high as 258.6 mg/g and a fast adsorption rate of 100 mg/L Cr^4+^ (concentration in about 20 min), indicating higher advantages than the current 2D graphene-based adsorbents and some other prevalent adsorbents. In another case, a water-soluble polyacrylic acid (PAA)/Fe_3_O_4_/GO nanocomposite was proposed by Zhang et al. [163] for the recyclable elimination of Pb^2+^, Cd^2+^ and Cu^2+^ ions from wastewater samples with extraordinary removal capacity and over 85% removal efficiency after five cycles.

**Figure 5:**
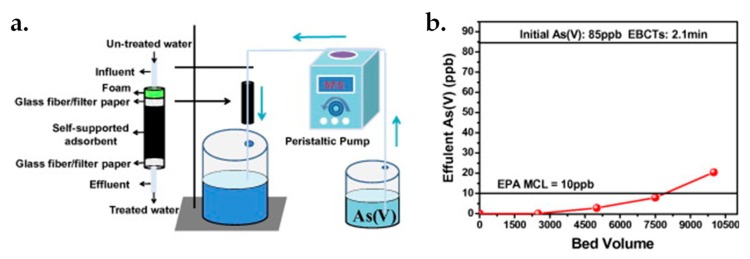
(**a**) Scheme of the As^5+^ removal from water by using Fe_2_O_3_/rGO hybrids as the filter. (**b**) Relations between bed volume and remaining effluent As^5+^ in wastewater. Reproduced from [164], with permission from Elsevier, 2014.

The high-efficient removal of heavy metal arsenic As^5+^ from contaminated water has also been demonstrated via 3D GBHMs. For example, Li et al. [164] fabricated a 3D bulk Fe_2_O_3_/rGO hybrid directly by on-site of GO reduction and Fe_2_O_3_ NPs deposition through a hydrothermal process. The hybrid was adopted to remove As^5+^ from water, as illustrated in Figure 5a. The amount of bed volumes at a threshold point (e.g., 10 ppb concentration of As^5+^) attained around 8000 with the initial As^5+^ concentration is 85 ppb (Figure 5b), indicating an excellent adsorption ability of the Fe_2_O_3_/rGO hybrids. It was found that, the surface complexation of metal oxyhydroxides and arsenic ions endows the Fe_2_O_3_/rGO adsorbent with strong As^5+^ removal ability. In addition, the proposed Fe_2_O_3_/rGO adsorbent could effectively inhibit the diffusion of Fe_2_O_3_ NPs into the water, thereby showing distinctive capability for water purification application. Furthermore, Fe_2_O_3_ possesses good performance in treating various heavy metals contaminated water such as As^5+^, As^3+^, Cu^2+^, Cr^4+^, Pb^2+^, etc. The verified high As^5+^ removal performance of the proposedFe_2_O_3_/rGO adsorbent provides a possibility for the elimination of other heavy metals under suitable conditions.

### 4.2. Removing Anions

The removal of anions in aqueous solution can be achieved through specific and/or nonspecific adsorption with various adsorbents [165]. The specific adsorption is associated with ligand exchange reactions in which the hydroxy groups in adsorbents were replaced by anions in wastewater, while the nonspecific adsorption is linked to the electrostatic interactions and primarily based on the pH of the adsorbent [166]. A decrement of pH in aqueous solution results in the electropositive surface, being enhanced by the electrostatic attraction between the adsorbent and the anions, thereby improving the removal efficiency of anionic pollutants [165].

Recently, titania-functionalized GO (T-F/GO) hybrid materials were synthesized and utilized as highly efficient adsorption materials to remove phosphate ions (PO_4_^3^¯) from wastewater. Owing to the synergistic effects between titania and GO in the PO_4_^3^¯ adsorption, T-F/GO hybrids exhibited much higher adsorption capacity (33.11 mg/g) than those of both pure titania and GO. Moreover, the decrement of pH enhanced the absorption capacities due to the increased attraction between phosphate anions and the charges on the T-F GO surface at low pH. Meanwhile, the addition of sodium ions was verified to increase the adsorption capacities.

Perchlorate (ClO_4_¯) pollutants are now worldwide issues impacting water system due to their strong solubility and non-reactivity, which can remain in water for long period of time and is hard to remove through the conventional separation techniques [167]. Graphene-based 3D materials provide a potential solution to solve this problem. For example, Zhang and co-workers [167] reported the synthesis of a graphene/polypyrrole (Ppy) composite by employing graphene nanosheets as the staging, which can be served as an ion exchanger for removing ClO_4_¯ from wastewater. The graphene/Ppy composite exhibited a significantly increased ingestion capacity for ClO_4_¯ compared than that of the sole Ppy film due to its 3D porous structure, which facilitated the dispersion of ClO_4_¯ into the Ppy film. Moreover, the existing of graphene matrix enhanced the stability of Ppy film in the operation process.

The removal of anionic dyes by employing 3D graphene-based materials has also been extensively studied [99,168,169]. For instance, Poly(diallyldimethylammoniumchloride)/GO (PDDA/GO) hydrogels were prepared by Wang et al. [169] and employed as high-efficient adsorbents to remove two kinds of anionic dyes, i.e., trypan blue (TB) and ponceau S (PS), from water pollution. The as-prepared PDDA/GO hydrogels offered very high adsorption efficiencies for TB and PS (>98%), owing to the strong π-π stacking and the anion-cation interaction. Meanwhile, after four adsorption and desorption cycles, the PDDA/GO hydrogels still maintained high adsorption capability towards these two anionic dyes.

### 4.3. Removing Organic Dyes

Organic dyes, such as methylene blue (MB), malachite green (MG), procion red MX-5B, rhodamine B (RB) and crystal violet (CV) are toxic and hazardous organic pollutants that originate from industrial branches including dyes manufacturing, textile finishing, paper printing and cosmetics in terms of effluent emission [170]. The release of untreated organic dyes into the water system has now become a serious global issue. It was reported that more than 700,000 tone dyes around the world are produced annually [171]. Due to the recalcitrant aromatic structures, the property of being resistant to aerobic digestion as well as antioxygenic characteristic of organic dyes [172], they are very stable and are very difficult to be treated from contaminated water via standard wastewater treatment facilities. To date, a large variety of treatment technologies such as adsorption [168], photocatalysis [173], biodegradation [174], etc. have been studied as ways to be eco-friendly and economically remove the organic dyes from wastewater. Besides, a number of materials are adopted by coupling with existing technologies for organic dye removal. Among them, carbon-based nanomaterials such as porous graphitic carbon [175] and CNTs [176] have been proved to be capable of exhibiting remarkable removal capacities for organic contaminates especially dyes. These engineered carbon materials, however, suffer from complex and cost-ineffective fabrication, poor dispersibility, and catalyst impurities [177].

Recently, 3D GBHMs have been attracting extensive attention on organic dyes removal, owing to their remaining excellent features of graphene and extension of the application potential of graphene, meanwhile, the integrated morphology endows them light for handling and being expedient for the subsequent separation process in real applications [178]. A list of publications on effective removal of various organic dyes in aqueous solution by employing 3D GBHMs are presented in Table 1. For example, Mei et al. [179] fabricated a series of 3D GA-zinc oxide (GA-ZnO) hybrid materials through a one-step hydrothermal synthesis in combination with a freeze-drying approach for eliminating MB in water. By assessing the adsorption capability and the performance of photocatalytic degradation in MB removal with the presence of light illumination, it was found that the prepared GA-ZnO hybrids possessed much improved absorptivity and stronger photocatalytic activity comparing to traditional ZnO and relevant materials with a best total MB removal efficiency as high as 97.6%. Ma et al. prepared the 3D porous structure GO/chitin nanofibrils (CNF) composite foams by incorporating CNFs into GO substrate, and adopted the fabricated GO-CNF foams for removing MB dyes from wastewater through batch column adsorptions, respectively [180]. Their results revealed that the removal efficiency of MB under the column adsorption process was very high, up to 98.5%, and was still maintained at around 90% even underwent three reuse rounds. Aside from organic dyes like MB, the GO-CNF material was demonstrated to be capable of eliminating other pollutants such as heavy metals and aromatics at extremely low concentrations in wastewater.

**Figure 6:**
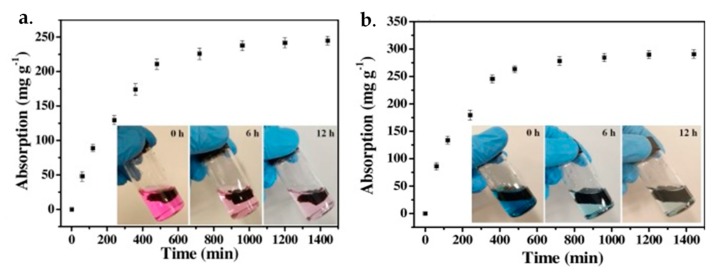
Applications of xanthan gum/graphene oxide (XG/GO-1) hybrid aerogels for organic dyes removal: (**a**) Adsorption of rhodamine B (RB). (**b**) Adsorption of methylene blue (MB). Reproduced from [183], with permission from Elsevier, 2017.

Shi et al. [181] reported a Ni and S co-doped 3D graphene hydrogels (N/S-GHs) by employing glutathione as the binding and reductive agents, and Ni and S as the modifying agents. The synthesized N/S-GHs were experimentally verified to possess outstanding performance towards the elimination of organic dyes including MB, MG, and CV in aqueous solution, allowing environmental-friendly preparation of 3D nanomaterials for organic wastewater treatment. In another case, Yusuf et al. [182] utilized a graphene-based 3D hybrid material by reducing GO and intercalating with cetyltrimethylammonium bromide (CTAB) towards the removal of azo dyes, such as acid orange 7 (AO7) and acid red 265 (AR265) from aqueous solution. The removal efficiency of 87.8% and 81.4% for AO7 and AR265 was obtained, respectively, in the first adsorption cycle of the fixed-bed column adsorption process, and the adsorption was still maintained at 67.7% and 61.5%, with 22.9% and 24.4% loss in AR265 and AO7 removal, respectively, after five consecutive adsorption and desorption rounds. The superior removal performance and good re-usability of GN-CTAB for dyes exhibited huge potential in the practical applications for wastewater treatment. Liu et al. fabricated a series of facile 3D xanthan gum (XG)/GO hybrid aerogels and used them to eliminate organic dyes from water [183]. By employing XG/GO hybrid aerogel, adsorption capacities as high as 244.36 mg/g and 290.57 mg/g for RB and MB dyes were obtained, respectively (Figure 6). Meanwhile, the adsorption capacity can be further improved by adjusting concentrations of GO within the hybrids.

### 4.4. Removing Drugs and Biomolecules

Pharmaceutical wastewaters are mostly toxic to both the environment and human beings [184]. Besides techniques such as photocatalysis, biodegradation, and ozonation [185], adsorption has recently been adopted to remove pharmaceuticals from contaminated water. Among various adsorption materials, graphene-based composite materials are known as the most promising ones for pharmaceutical wastewater treatment and they have received increasing attention.

Kyzas et al. [186] demonstrated the synthesis of GO/PAA-grafted chitosan nanocomposite (GO/CSA), which can utilized to remove dorzolamide pharmaceutical compound (an active pharmaceutical ingredients) from biomedical synthetic wastewaters. The adsorption behavior is related to the specific surface features of GO/polymer nanocomposite and the adsorption mechanism is ascribed to the interactions between active groups of the GO or CSA and aromatic groups of the dorzolamide. The created GO/CSA hybrids exhibited stronger adsorption capability (334 mg/g) than that of pure GO (175 mg/g) and CSA (229 mg/g), respectively, due to the presence of a large amount of –COOH groups in the GO/CSA composite. A recently published study demonstrated the adsorptive removal of anti-inflammatory drugs (AIDs), i.e., naproxen (NAP) and ketoprofen (KTP), from wastewater using highly porous GO/metal-organic framework (GO/MOF) composites. The GO/MOF composites were synthesized by combining MIL-101(Cr) with GO. The as-prepared GO/MIL-101 hybrids displayed highly improved adsorption towards both NAP and KTP compared to commercial AC and pristine MIL-101, as well as some other frequently adopted adsorbents, owing to H-bonding with the existed several functional groups in composites. The regeneration of GO/MIL-101 composite can be easily achieved by ethanol washing without severe degradation, endowing it a fascinating adsorbent for AIDs elimination from aqueous solution.

Hydroxyl- and amino-substituted aromatics are ionizable aromatic compounds that are commonly found in effluents from the pesticides and pharmaceutical related industries. The effective removal of those aromatic pollutants from contaminated water has attracted considerable attention. The utilization of iron oxide NPs-decorated rGO hybrid materials to eliminate 1-naphthol and 1-naphthylamine from aqueous solution was reported by Yang and co-workers [187]. The adsorption experiments revealed that the synthesized rGO/iron oxide materials are capable of effectively adsorbing 1-naphthylamine and 1-naphthol and they can be easily recovered through magnetic separation.

Glyphosate, an organophosphorus pesticide, was applied widely as herbicides in the agricultural field. Nevertheless, contaminated water with glyphosate is identified as a severe worldwide issue, which has attracted extensive attention [188]. Yamaguchi and co-workers [188] reported the fabrication of the 3D graphene-based hybrid composites by immobilizing MnFe_2_O_4_ microspheres onto rGO nanosheets (MnFe_2_O_4_-G) via a facile one-step solvothermal process. The prepared MnFe_2_O_4_-G was further used as an adsorbent for removing glyphosate from water. It was found that the hybrids hold a specific area as large as 305.5 m^2^/g as the microspheres were uniformly distributed onto the graphene nanosheets. The adsorption of glyphosate on MnFe_2_O_4_-G depends mainly on the mutual effect of the electrostatic interaction and the ion exchange, consequently, a maximum glyphosate adsorption capability of 39 mg/g was achieved.

### 4.5. Oil-Water Separation

Water pollution attributed to the increasing occurrences of industrial oily wastewater emissions and oil spills is now a worldwide issue which affects global environment and ecosystems. Effective oil-water separation has become an urgent task towards environmental protection and the sustainable development of the society [189].

**Figure 7:**
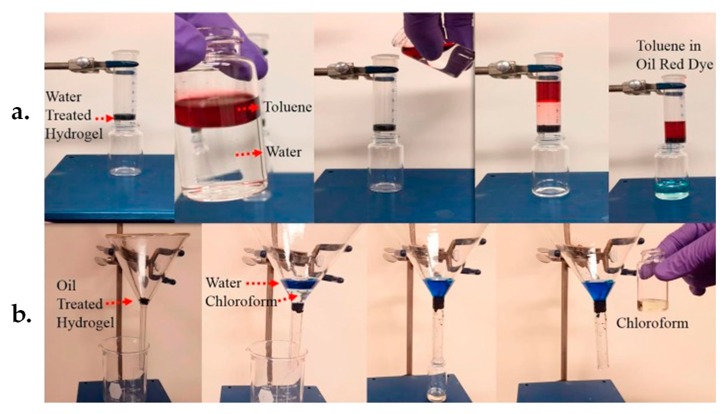
Oil-water separation employing (**a**) LA/F/rGO hydrogel pretreated with water for oil (toluene) separation from water, and (**b**) LA/F/rGO hydrogel pretreated with oil for water separation from oil (chloroform). Reprinted with the permission from ref. Reproduced from [191], with permission from Elsevier, 2018.

Recently, 3D graphene-based hydrogel materials have received widespread attention in oil-water separation [190]. In view of water or oil separation from solutions, Hu et al. [191] reported the synthesis of amphiphobic LA/F/rGO hydrogels through a hydrothermal process and adopted them for oil-water separation. The as-prepared LA/F/rGO hydrogels exhibited outstanding performance in oil-water separation, particularly, allowing selective and high-efficient filtration of oil or water owing to both oil and water resistance surface features. It was experimentally demonstrated that the proposed LA/F/rGO hydrogel allows water to pass through while prohibits toluene (act as one model oil) flow via water pretreating (Figure 7a), resulting in a successful separation of oil from water. In addition, it allows chloroform (act as another model oil) to pass through while prohibits water flow via oil pretreating (Figure 7b), allowing for a successful separation of water from oil.

**Figure 8:**
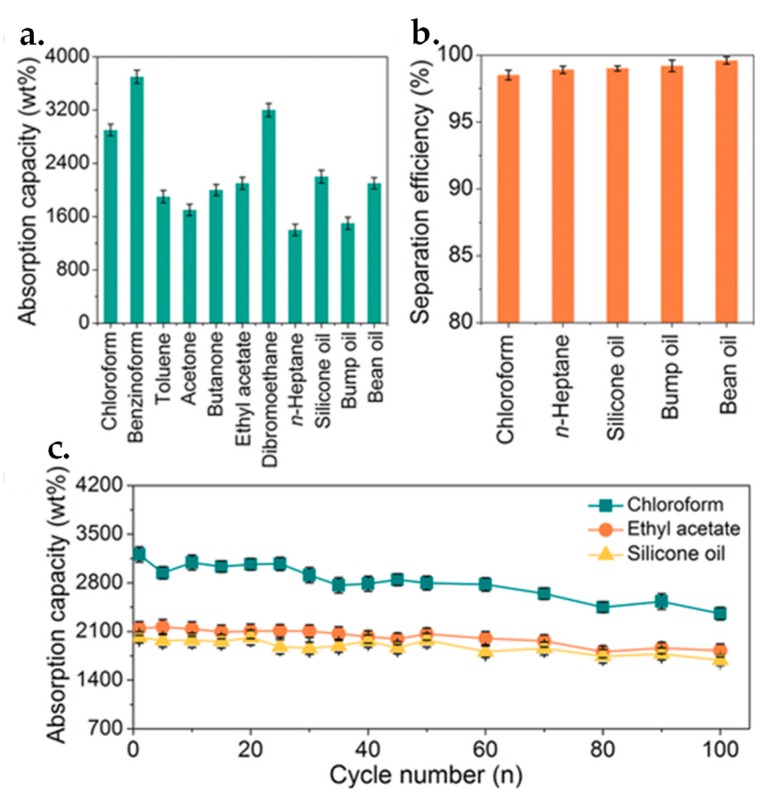
Absorption and separation performances of the ZIF-8@rGO@Sponge on various oils and organic solvents: (**a**) Absorption capacity. (**b**) Separation efficiency. (**c**) Absorption recyclability. Reproduced from [195], with permission from Wiley, 2019.

Besides, 3D graphene/MOF-based composite materials were recently explored for oil-containing wastewater disposal in view of the inherent characteristics of graphene such as high porosity, ultralow density, and excellent mechanical strength [192]. More importantly, the integration of MOFs with graphene prevents the agglomeration and overlapping of graphene nanosheets, possessing superior wettability and amphiphobic features towards oil and water [193,194], thereby enabling improved performance of 3D graphene/MOF-based materials in oil-water separation. For instance, Gu et al. developed the wrinkled 3D MOF@rGO microspheres with superhydrophobic and superoleophilic properties by intercalating crumpled rGO nanosheets with zeolitic imidazolate frameworks (ZIF-8) NPs [195]. The synthesized ZIF-8@rGO microspheres were then assembled into a commercial PU sponge via dip-coating and thermal crosslinking and utilized as the absorbents for oil-water separation. The as-prepared ZIF-8@rGO@Sponge exhibited prominent separation (separation efficiency above 98%) and adsorption (absorption capacity of 1400–2900 wt.% depending on different oils) performances and good recyclability (slight absorption capacity drop after 100 cycles) on a number of organic solvents and oils from aqueous solution (Figure 8), and the adsorbents still maintained high mechanical strength and superhydrophobicity properties even with multiple extrusion. Their work provides a promising perspective towards the development of graphene/MOF-based composites on oil-water separation and wastewater treatment.

### 4.6. Seawater Deionization

The scarcity of clean freshwater has been becoming a global problem due to the growing population and environmental pollution. Seawater desalination is known as a key solution to address this issue. Traditional techniques based on membrane desalination such as reverse osmosis [196], electric desalination (like electrodialysis) [197], and thermal distillation including multistage flash evaporation [198], have been well developed towards effective desalination of saline water. However, these techniques suffer from ongoing problems including expensive equipment manufacturing, high energy consumption and/or membrane fouling [199]. Graphene-based nanomaterials, which provide the opportunity of integrating other nanomaterials or incorporating them into a host substrate to form nanocomposites, are capable of merging various features into a single material [200]. Exploiting graphene-based nanocomposites as membranes [201,202,203], with their desired high permeate flux and strong antifouling capability, are expected to offer a solution for overcoming the bottlenecks of current desalination technologies [204].

The synthesis of rGO/polyaniline (rGO-PANI) composite material has been reported through the conjugation of rGO with PANI via oxidative polymerization. The rGO-PANI composites were served as a nano-filler in PSf membranes and were utilized for desalination [201]. Hydrophobic membrane surfaces with expanded porosity and enlarged macropores were achieved due to the incorporation of rGO into the membrane substrate, thereby improving the pure water flux comparing to the bare PSf membrane. Consequently, the fabricated membranes exhibited an enhanced salt rejection (82% at 10 bar) with 0.5 wt.% rGO/PANI usage.

**Figure 9:**
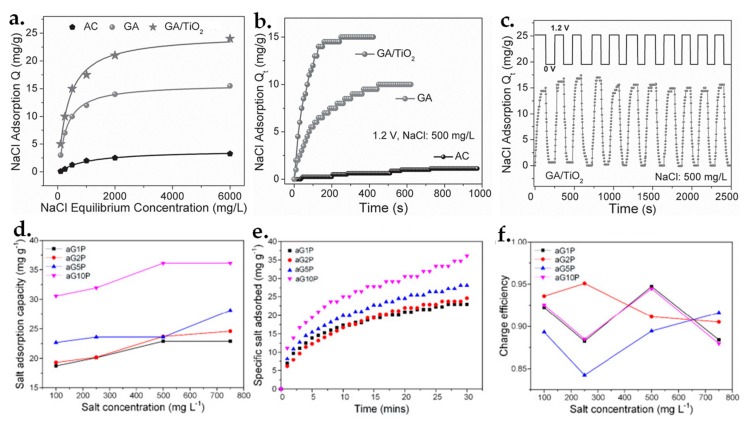
Desalination performances of three-dimensional (3D) GA/TiO_2_ hybrids and graphene oxide (GO)-polyvinyl alcohol (PVA) composites as CDI and M-CDI electrode materials: (**a**) Salt adsorption capacity of activated carbon (AC), graphene aerogel (GA) and GA/TiO_2_. (**b**) Time dependent desalination behaviors of AC, GA and GA/TiO_2_. (**c**) Desalination and regeneration cycles of GA/TiO_2_. (**a**–**c**) were reproduced from [211], with permission from Wiley, 2013. (**d**) Salt adsorption capacity as functions of salt concentration for four different GO:PVA ratios (G*n*P, *n* = 1,2,5 or 10). (**e**) Specific salt adsorption at a NaCl concentration of 750 ppm. (**f**) Dependent of charge efficiency on different salt concentrations. (**d**–**f**) were reproduced from [217], with permission from Elsevier, 2019.

In the past years, CDI has emerged as a promising alternative technology for seawater deionization owing to energy saving, low process costs and ease of maintenance [205,206,207]. To achieve high desalination capacity and salt removal efficiency in CDI process, 3D GBHMs are often employed as porous electrodes since graphene has its unique characteristics [208,209,210]. Moreover, the incorporation of graphene sheets into 3D networks endows a huge quantity of open pores for salt ions in seawater and enables various functional inorganic nanomaterials to be constructed [211,212,213,214]. For example, Yin and co-workers [211] reported the fabrication of 3D graphene/metal oxide NP hybrids, which were employed as CDI electrode materials for the deionization of saline water. The synthesized G/TiO_2_ hybrids presents higher electroadsorption capacity towards NaCl, faster electroadsorption rate, and better reversibility (Figure 9a–c), comparing to pure GA and commercial AC, as well as some other reported CDI electrode materials [149,215,216].

In addition to TiO_2_, the electrode capacitance can also be enlarged by the integration of other metal oxides in graphene-based materials [218,219]. In a recent report [219], El-Deen et al. proposed the preparation of graphene/MnO_2_ hybrids by embedding MnO_2_ into graphene nanosheets. The fabricated graphene/MnO_2_ nanorod hybrids were utilized as high-performance CDI electrodes for desalination. The electrosorption capacity was enhanced owing to the isolation of graphene adjacent layers through the sandwiched MnO_2_ particles to prohibit sheet overlap. Meanwhile, MnO_2_ particles enlarged the contact area with graphene sheets to retain more salt ions on both the electrode surface and in/between the graphene sheets. Consequently, the synthesized graphene/MnO_2_ nanorod revealed outstanding deionization performance including adsorptive capacity (5.01 mg/g), specific capacitance (292 F/g), salt removal efficiency (up to 93%) and recyclability, outperforming many reported CDI electrode materials.

To improve both removal and energy efficiencies of the seawater desalination process, derived CDI systems such as membrane-based CDI (MCDI) [220,221] and flow electrode CDI (FCDI) [222,223] have been developed recently by utilizing 3D graphene-based materials [217,224,225]. A very recent study demonstrated [217] the adoption of a novel 3D structured AC material by cross-linking GO with PVA and then activating by potassium hydroxide as MCDI cell, in combination with additional ion-exchange membranes for saline water desalination. The desalination performance of the proposed 3D GO-PVA composites in a MCDI configuration was evaluated through specific salt adsorption, salt adsorption capability and charge efficiency, respectively. A salt adsorption capability as high as 36.1 mg/g in 750 mg/L salt solution was obtained with 1.2 V applied voltage (Figure 9d,e) and the corresponding charge efficiencies (Figure 9f) reported by Leong and co-workers are higher than that of conventional CDI [226] and MCDI using porous carbons [227].

## 5. Conclusions and Outlooks

In summary, 3D GHBMs exhibited wide applications in water purification applications. Several typical fabrication techniques, such as template-based synthesis, self-assembly, ice-drying of hydrogels, and 3D printing are highly effective to prepare 3D GBHMs with adjustable structure and properties. With the fabricated 3D GBHMs, some water purification methods including filtering, adsorption/removal, photocatalytic degradation, electrocatalytic degradation, and bio-degradation are useful for removing various metallic ions, anions, organic dyes, drugs, biomolecules, oils, and others from the polluted water systems. It is clear that the porous structure and the conjugated hybrid nanomaterials of 3D GBHMs play important roles in the water purification performance. Compared to the traditional 2D organic and inorganic membranes/films, 3D GBHMs reveal better performance towards the water purification process.

Although great achievements have been obtained on the fabrication of 3D GHBMs for water purification applications recently, in our opinion, there are still spaces in this field need to be filled in. For instance, firstly, the large-scale production of 3D GHBMs with high efficiency should be developed. The self-assembly and hydrothermal synthesis of 3D GHBMs in solution could be the potential way to achieve in this aim. Second, the practical applications of 3D GHBMs for the water streams treatments should be further investigated, in which the economic production of these materials for stable, recyclable, and environment-adaptable water purification are highly expected. Third, the pores of 3D GHBMs should be adjustable in the nanoscale range to obtain higher water purification ability. In addition, the mechanical stability of 3D GHBMs should be considered by adding chemical linker to improve their re-usability. Fourth, it is necessary to evaluate the antifouling ability of 3D GHBMs such as proteins, peptide, virus, and other biomolecules could bind onto the surface of materials, blocking the pores in materials and decreasing the purification ability of 3D GHBMs. Therefore, more efforts should be made to investigate the mutual effects between various biomolecules and the surface of materials, and to explore new surface modification techniques to inhibit the biofouling. Fifth but not the last, it is possible to conjugate graphene materials with some biopolymers and biomacromolecules to form biological 3D GHBMs, which could reveal boarder applications in biomedical and tissue engineering communities.

## Figures and Tables

**Table 1:** List of 3D GBHMs utilized for eliminating organic dyes in aqueous solution.

Adsorbent	Organic Dye	Dye Concentration [mg/L]	Adsorption Performance	Reference
3D GO/DNA composite hydrogels	Safranine O (SO) dye	100	Nearly 100% removal efficiency in 24 h; 960 mg/g dye loading capacity	[86]
3D GA-ZnO hybrids	MB dye	20	Up to 97.6% removal efficiency in 3 h	[179]
3D GO-CNF composite foams	MB dye	30	Up to 98.5% removal efficiency in 4 h	[180]
3D N/S-GHs hydrogels	MB, MG, and CV dyes	140	Superior removal efficiency in 10 h; adsorption capacities of 738.1 mg/g for MG, around 625 mg/g for MB and around 600 mg/g for CV	[181]
3D GN-CTAB composites	AO7 and AR265 dyes	100	92.18% and 89.13% removal efficiency for AR265 and AO7, respectively in 3 h; adsorption capacities of 511 mg/g and 356 mg/g for AR265 and AO7, respectively	[182]
3D XG/GO hybrid aerogels	RB and BM dyes	100	Around 95% and 96% removal efficiency for RB and MB, respectively in 12 h; adsorption capacities of 244.36 mg/g and 290.57 mg/g for RB and MB, respectively	[183]
